# Albuminuria is associated with higher turnover-related bone histomorphometric indices in chronic kidney disease stages 3–4

**DOI:** 10.1093/ckj/sfag346

**Published:** 2026-10-01

**Authors:** Ricardo Neto, Bernardo Fernandes, Juliana Magalhães, Inês Alencastre, João Frazão

**Affiliations:** RISE-Health, Faculty of Medicine, University of Porto, Porto, Portugal; Department of Medicine, Faculty of Medicine, University of Porto, Porto, Portugal; Department of Nephrology, ULS São João, Porto, Portugal; RISE-Health, Faculty of Medicine, University of Porto, Porto, Portugal; Department of Nephrology, ULS São João, Porto, Portugal; Department of Medicine, Faculty of Medicine, University of Porto, Porto, Portugal; USF Barcel Saúde, ULS Barcelos/Esposende, Barcelos, Portugal; RISE-Health, Faculty of Medicine, University of Porto, Porto, Portugal; Department of Medicine, Faculty of Medicine, University of Porto, Porto, Portugal; RISE-Health, Faculty of Medicine, University of Porto, Porto, Portugal; Department of Medicine, Faculty of Medicine, University of Porto, Porto, Portugal; Department of Nephrology, ULS São João, Porto, Portugal

**Keywords:** albuminuria, bone biopsy, bone histomorphometry, chronic kidney disease, renal osteodystrophy

## Abstract

**Background:**

Biological determinants of bone turnover in chronic kidney disease (CKD) remain incompletely understood. Although albuminuria is an established marker of kidney damage and predictor of renal and cardiovascular outcomes, its relationship with bone remodeling, as assessed by quantitative bone histomorphometry, has not previously been investigated.

**Methods:**

We performed a cross-sectional study of 56 adults with CKD stages 3–4 who underwent transiliac bone biopsy with quantitative histomorphometry following double tetracycline labeling. Associations between 24-h urinary albumin excretion and individual bone histomorphometric parameters were evaluated using multivariable linear regression. Models were adjusted for age, sex, estimated glomerular filtration rate (eGFR), intact parathyroid hormone (iPTH), and fibroblast growth factor 23 (FGF23).

**Results:**

In multivariable analyses, higher 24-h urinary albumin excretion was associated with a histomorphometric pattern consistent with increased bone turnover, reflected by greater bone formation and cell surface indices. Specifically, log₁₀-transformed urinary albumin excretion was associated with higher bone formation rate (*B* = 0.295, 95% CI 0.088 to 0.501; *P* = 0.008), osteoblast surface (*B* = 0.588, 95% CI 0.187 to 0.988; *P* = 0.005) and osteoclast surface (*B* = 0.464, 95% CI 0.041 to 0.887; *P* = 0.033). Associations with mineralization-related parameters and bone volume were not statistically significant.

**Conclusions:**

In patients with CKD stages 3–4, higher urinary albumin excretion was independently associated with quantitative histomorphometric indices of higher bone turnover. These findings suggest that albuminuria may mark a bone remodeling phenotype not fully captured by eGFR, iPTH, and FGF23. Larger prospective studies are needed to define the biological basis and clinical implications of this association.

## Graphical Abstract

**Graphical Abstract:**
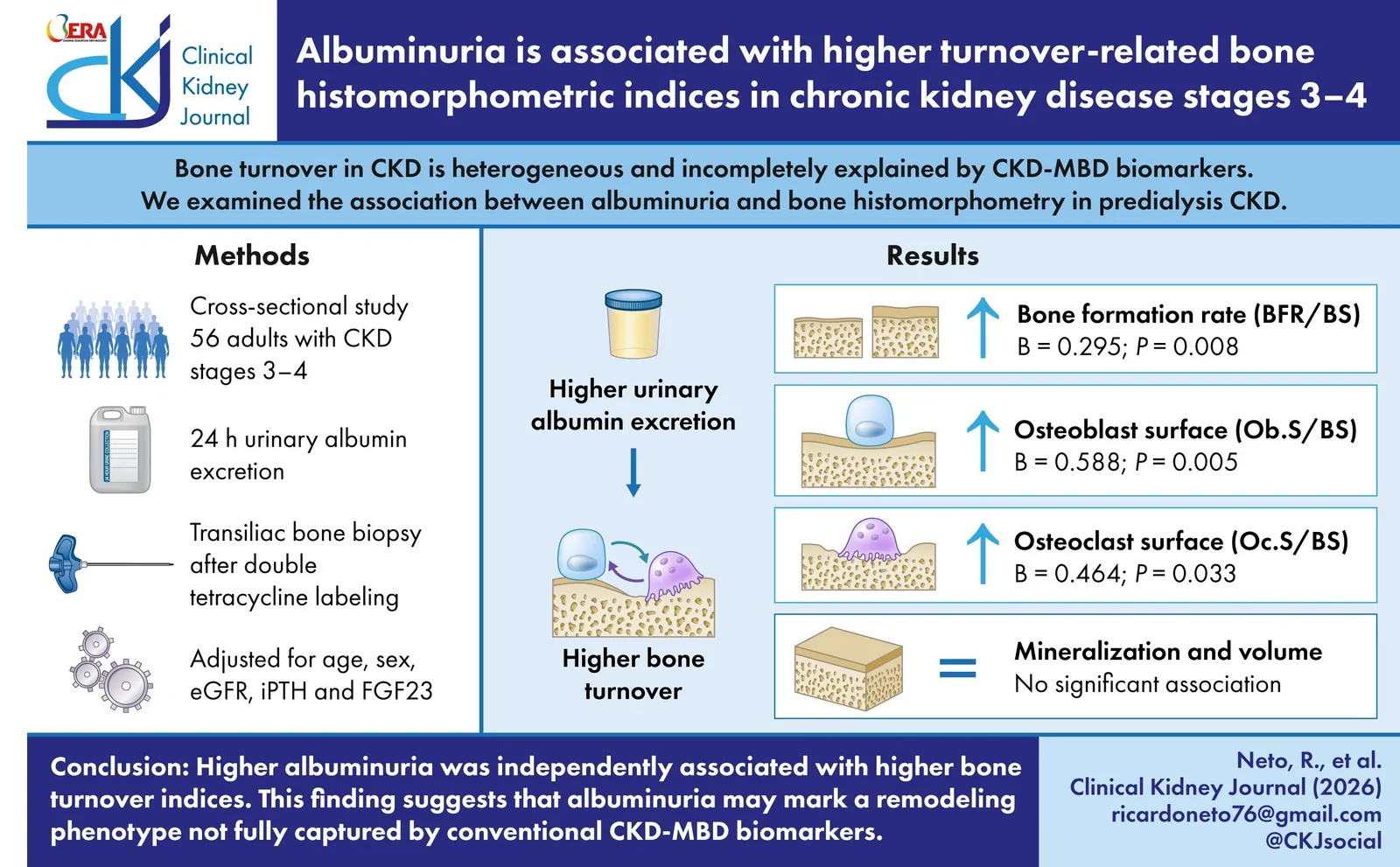

KEY LEARNING POINTS **What was known:**

- Bone turnover in chronic kidney disease (CKD) is heterogeneous and incompletely explained by conventional CKD-MBD biomarkers.
- Albuminuria is an established marker of kidney damage and adverse renal, cardiovascular, and skeletal outcomes, but its relationship with bone remodeling assessed by quantitative bone histomorphometry has not been defined.

**This study adds:**

- In 56 adults with CKD stages 3–4 who underwent transiliac bone biopsy, greater 24-h urinary albumin excretion was independently associated with higher bone formation rate (BFR/BS).
- Greater urinary albumin excretion was also associated with increased osteoblast surface and osteoclast surface after adjustment for age, sex, estimated glomerular filtration rate (eGFR), intact parathyroid hormone (iPTH), and fibroblast growth factor 23 (FGF23).
- No significant associations were observed with mineralization-related parameters or bone volume.

**Potential impact:**

- Albuminuria may mark a bone remodeling phenotype not fully captured by eGFR, iPTH, and FGF23.
- These findings support larger prospective studies to determine whether albuminuria has clinical value in identifying CKD patients with altered skeletal remodeling.

## INTRODUCTION

Bone remodeling varies substantially among patients with chronic kidney disease (CKD), yet the biological determinants of this variability remain poorly understood [1–3]. Patients with similar degrees of kidney dysfunction may exhibit low-, normal- or high-turnover bone disease, suggesting that factors beyond glomerular filtration rate contribute to skeletal phenotype. This biological heterogeneity is a defining feature of chronic kidney disease–mineral and bone disorder (CKD-MBD) but remains incompletely explained by conventional clinical and biochemical parameters [4–7].

Albuminuria is a well-established marker of kidney damage and an independent predictor of CKD progression and cardiovascular events [8–10]. Beyond its prognostic significance, proteinuric kidney disease has been linked to disturbances of mineral metabolism, including altered vitamin D homeostasis, fibroblast growth factor 23 (FGF23)–Klotho signaling, phosphate handling, and other electrolyte abnormalities [11–17]. These observations support the concept of an integrated endocrine network linking the kidney, bone, and vasculature in CKD-MBD [4, 7, 18].

Consistent with this framework, previous clinical and experimental observations have linked proteinuria to abnormalities in bone histology and bone cell activity [19–22]. However, whether urinary albumin excretion is associated with quantitative bone histomorphometric parameters in predialysis CKD has not been investigated. Bone histomorphometry remains the reference standard for evaluating renal osteodystrophy and is particularly suited to address this question because it directly quantifies bone turnover rather than relying on circulating surrogate biomarkers [23–29].

We therefore investigated the association between 24-h urinary albumin excretion and bone histomorphometric parameters in patients with CKD stages 3–4 who underwent transiliac bone biopsy.

## MATERIALS AND METHODS

### Study design and participants

This was a secondary cross-sectional analysis of a previously characterized 56-patient predialysis CKD biopsy cohort. Adults with CKD stages 3–4 were consecutively recruited from the predialysis outpatient clinic of ULS São João between February 2014 and September 2019. Eligible participants were aged ≥18 years and had an estimated glomerular filtration rate (eGFR) between 15 and 60 ml/min/1.73 m². Exclusion criteria were previous parathyroidectomy, autoimmune or chronic inflammatory disease, active malignancy, impaired ambulation, and previous treatment with medications known to affect bone metabolism, including calcium or aluminum salts, native or active vitamin D, glucocorticoids, and bisphosphonates. No additional participants from the original cohort were excluded from descriptive analyses. The study was approved by the local Ethics Committee (CES 299–13), and all participants provided written informed consent.

### Clinical and laboratory assessment

Baseline clinical data included age, sex, body mass index, CKD etiology, diabetes mellitus, blood pressure, and use of renin–angiotensin–aldosterone system inhibitors. Fasting blood samples and 24-h urine collections were obtained at the time of bone biopsy. Biochemical measurements, including serum creatinine, calcium, phosphorus, albumin, bicarbonate, alkaline phosphatase, iPTH, 25-hydroxyvitamin D [25(OH)D], and FGF23, were performed as previously described [30]. eGFR was calculated using the Chronic Kidney Disease Epidemiology Collaboration (CKD-EPI) equation [31]. CKD stages were classified according to the Kidney Disease: Improving Global Outcomes (KDIGO) Clinical Practice Guideline for the Evaluation and Management of Chronic Kidney Disease [32]. Adequacy of urine collections was assessed using urinary creatinine excretion.

### Bone biopsy and histomorphometric analysis

Transiliac bone biopsies were performed after double tetracycline labeling (doxycycline 200 mg twice daily for 3 days, repeated after a 12-day interval) using a modified Bordier trephine, as previously described [30]. Undecalcified bone specimens were embedded in methyl methacrylate. Five-micrometer sections were stained with modified Masson–Goldner trichrome for static histomorphometric analysis, whereas unstained 10-μm sections were used for dynamic fluorescence analysis. Approximately 30 consecutive fields per section were analyzed at ×20 magnification using OsteoMeasure software (OsteoMetrics, Decatur, GA, USA). Bone histomorphometric analyses were performed independently by two observers experienced in renal bone histomorphometry and blinded to all clinical and biochemical data.

Static and dynamic histomorphometric parameters were quantified according to the nomenclature of the American Society for Bone and Mineral Research [25]. Bone histology was classified according to the turnover, mineralization, and volume system of renal osteodystrophy [6]. Reference ranges were derived from previously published normative studies and clinical practice guidelines [33–36]. Low and high bone turnover were identified using bone formation rate/bone surface (BFR/BS) cut-offs of <10.95 and >37.5 µm³/µm²/yr, respectively. Normal mineralization was defined as mineralization lag time (Mlt) <50 days, and low bone volume as bone volume/tissue volume (BV/TV) <16%. The single participant with mixed uremic osteodystrophy was excluded from phenotype-based comparisons.

### Statistical analysis

Continuous variables are presented as mean (standard deviation) or median (interquartile range), according to their distribution, whereas categorical variables are presented as frequencies and percentages. Comparisons among bone histological phenotypes were performed using Kruskal–Wallis test for continuous variables and Fisher’s exact test for categorical variables.

Univariable and multivariable linear regression models were used to evaluate the association between 24-h urinary albumin excretion and individual quantitative bone histomorphometric parameters. BFR/BS was considered the primary turnover-related histomorphometric parameter; analyses of other histomorphometric indices were considered exploratory. Adjusted models included age, sex, eGFR, log₁₀-transformed intact parathyroid hormone (iPTH), and log₁₀-transformed FGF23. Additionally, a sequential adjustment analysis was performed for the primary outcome (BFR/BS). Starting from the univariable model, eGFR, log₁₀(iPTH), log₁₀(FGF23), age, and sex were added incrementally to evaluate the robustness of the association between urinary albumin excretion and BFR/BS. All sequential models were fitted in the same complete-case sample (*n* = 27) to ensure comparability across models. This parsimonious adjustment set was selected *a priori* to account for demographic factors, kidney function, and major CKD-MBD biomarkers while limiting overfitting given the modest sample size.

Urinary albumin excretion, total urinary protein excretion, iPTH, FGF23, and all histomorphometric parameters were log₁₀-transformed before analysis to reduce skewness and improve the distribution of model residuals. For histomorphometric variables containing zero values, log₁₀-transformation was performed after adding 0.001 to all values, a constant substantially smaller than the lowest non-zero values. Regression coefficients represent the expected change in the dependent variable per 1-unit increase in log₁₀ urinary albumin excretion. Unstandardized regression coefficients (B), 95% confidence intervals (CIs), and *P* values are reported. In sensitivity analyses, analogous multivariable models were fitted using total urinary protein excretion instead of urinary albumin excretion. Model assumptions were assessed using residual diagnostics. For the primary model, linearity was further assessed by including a quadratic term for log₁₀ urinary albumin excretion, and homoscedasticity was evaluated using the Breusch–Pagan test. Collinearity and influential observations were assessed using variance inflation factors, leverage and Cook’s distance. No imputation of missing data was performed. Dynamic histomorphometric parameters, including BFR/BS, required adequate double tetracycline labeling and interpretable fluorescent labels, and were therefore not quantifiable in all biopsies. Regression analyses were performed as complete-case analyses, with sample size varying across models because dynamic histomorphometric parameters and some biochemical covariates were not available for all participants.

Statistical analyses were performed using IBM SPSS Statistics version 31.0 (IBM Corp., Armonk, NY, USA). All tests were two-sided, and *P* values <0.05 were considered statistically significant.

## RESULTS

### Clinical, biochemical, and histomorphometric characteristics

The study population included 56 patients with CKD stages 3–4. Participants were predominantly male, with a high prevalence of diabetes. Biochemically, most patients had low vitamin D status, with substantial interindividual variability in circulating iPTH and FGF23 concentrations (Table 1).

**Table 1:** Clinical and laboratory characteristics according to bone histological phenotype.

	Normal bone histology (*n* = 23, 41.1%)	Low-turnover bone disease (*n* = 20, 35.7%)	High-turnover bone disease (*n* = 12, 21.4%)	*P* value
Clinical				
Age (yr)	67.3 (10.3)	66.4 (9.9)	63.3 (9.6)	0.215
Male (%)	69.6	90.0	75.0	0.278
Diabetes (%)	56.5	75.0	50.0	0.328
Body mass index (kg/m^2^)	28.9 (2.5)	29.4 (4.7)	26.7 (5.1)	0.163
CKD etiology (%)				0.993
Diabetes mellitus	30.4	30.0	33.3	
Hypertension	30.4	40.0	33.3	
Glomerular disease	21.7	20.0	16.7	
Other	17.4	10.0	16.7	
SBP (mmHg)	134 (9)	137 (12)	136 (14)	0.523
RAAS inhibitors (%)	78.3	88.9	66.7	0.285
Biochemistry				
Creatinine (mg/dl)	2.10 (0.34)^a^	2.30 (0.33)	2.71 (0.46)	0.001
eGFR (ml/min/1.73 m^2^)	29.9 (7.7)^a^	28.5 (5.5)	22.9 (5.3)	0.005
Hemoglobin (g/dl)	12.5 (2.1)	13.6 (1.4)	13.2 (1.3)	0.054
Albumin (g/dl)	4.08 (0.27)	4.22 (0.34)	4.28 (0.29)	0.099
Bicarbonate (mmol/l)	25.6 (3.3)	25.3 (3.4)	24.8 (3.4)	0.691
C-reactive protein (mg/L)	1.6 (0.9, 6.5)	3.4 (0.9, 7.5)	1.7 (0.8, 3.4)	0.406
Calcium (mg/dl)	9.4 (0.5)^a^	9.4 (0.3)^a^	9.1 (0.4)	0.021
Phosphorus (mg/dl)	3.6 (0.7)	3.4 (0.5)	3.7 (0.4)	0.202
25(OH)D (ng/ml)	15 (11, 22)	16 (13, 23)	15 (9, 22)	0.887
ALP (U/L)	79 (64, 91)	74 (52, 98)	99 (66, 144)	0.138
iPTH (pg/ml)	79.4 (45.2, 116.9)^a^	83.9 (51.3, 125.9)^a^	207.4 (126.3, 274.2)	0.012
FGF23 (pg/ml)	22.7 (9.4, 34.2)^a^	23.3 (16.3, 31.6)^a^	43.5 (28.2, 75.4)	0.010
Urinary protein (mg/day)	730 (270, 1870)	320 (162, 1330)	1470 (400, 3830)	0.070
Urinary albumin (mg/day)	212 (38, 1016)	81 (11, 593)	1717 (339, 3819)	0.052

SBP, systolic blood pressure; CKD, chronic kidney disease; RAAS, renin-angiotensin-aldosterone system; eGFR, estimated glomerular filtration rate calculated using the CKD-EPI equation; 25(OH)D, 25 hydroxyvitamin D; ALP, alkaline phosphatase; iPTH, intact parathyroid hormone; and FGF23, fibroblast growth factor-23.

Data are reported as mean (SD) for normally distributed variables, median (interquartile range) for non-normally distributed variables, or percentage for categorical variables. *P* values were calculated using Kruskal–Wallis test for continuous variables and Fisher’s exact test for categorical variables. Adequacy of 24-h urine collections was assessed using urinary creatinine excretion.

a *P* < 0.05 vs. high-turnover bone disease.

Histomorphometric analysis showed normal bone histology in 23 patients (41.1%), low bone turnover with normal mineralization (adynamic bone disease) in 20 (35.7%), and high bone turnover with normal mineralization (hyperparathyroid bone disease) in 12 (21.4%). The single participant (1.8%) with high bone turnover and abnormal mineralization, consistent with a mixed uremic osteodystrophy pattern, was excluded from phenotype-based group comparisons but was eligible for analyses of continuous histomorphometric parameters. No cases of low bone turnover with abnormal mineralization (osteomalacia) were observed.

Across bone histological phenotypes, most baseline clinical characteristics, including age, sex distribution, diabetes prevalence, body mass index, CKD etiology, and systolic blood pressure, were broadly similar. eGFR was lowest in patients with high-turnover bone disease, who also had lower serum calcium and higher circulating iPTH and FGF23 concentrations. Urinary albumin excretion was numerically highest in patients with high-turnover bone disease, although differences across histological groups did not reach statistical significance (*P* = 0.052).

Quantitative bone histomorphometric parameters according to bone histological phenotype are summarized in Table 2. Turnover-related indices showed a clear gradient across phenotypes, with formation and resorption parameters increasing progressively from low-turnover to high-turnover bone disease.

**Table 2:** Histomorphometric parameters according to bone histological phenotype.

	Normal bone histology (*n* = 23, 41.1%)	Low-turnover bone disease (*n* = 20, 35.7%)	High-turnover bone disease (*n* = 12, 21.4%)	*P* value
Turnover				
BFR/BS (µm^3^/µm^2^/yr)	17.43 (14.03, 20.75)^a,b^	6.10 (1.62, 9.70)^a^	39.97 (38.21, 43.07)	<0.001
Ob.S/BS (%)	0.53 (0.27, 0.80)^a,b^	0.04 (0, 0.17)^a^	2.06 (0.97, 2.39)	<0.001
Oc.S/BS (%)	0.26 (0.07, 0.45)^a,b^	0 (0, 0.09)^a^	0.72 (0.33, 0.97)	<0.001
OS/BS (%)	13.50 (9.19, 18.98)^a,b^	6.18 (3.60, 11.96)^a^	24.86 (20.75, 41.61)	<0.001
ES/BS (%)	2.79 (2.16, 4.02)^a,b^	1.10 (0.62, 2.21)^a^	8.19 (3.73, 11.04)	<0.001
Mineralization				
Mlt (d)	22.80 (15.53, 31.03)	18.29 (13.29, 39.47)	13.87 (12.58, 24.01)	0.274
OV/BV (%)	2.94 (2.19, 3.35)^b^	0.99 (0.52, 2.32)^a^	3.69 (2.64, 6.18)	<0.001
O.Th (µm)	7.13 (6.25, 7.74)^b^	5.07 (3.53, 6.01)^a^	6.68 (6.09, 7.99)	0.001
Volume				
BV/TV (%)	18.18 (16.67, 24.99)	16.22 (10.79, 20.29)^a^	22.59 (18.65, 25.14)	0.005

BFR/BS, bone formation rate/bone surface; Ob.S/BS, osteoblast surface/bone surface; Oc.S/BS, osteoclast surface/bone surface; ES/BS, eroded surface/bone surface; OS/BS, osteoid surface/bone surface; Mlt, mineralization lag time; OV/BV, osteoid volume/bone volume; O.Th, osteoid thickness; BV/TV, bone volume/tissue volume.

Data are reported as median (interquartile range). *P* values were calculated using the Kruskal–Wallis test.

a *P* < 0.05 vs. high-turnover bone disease.

b *P* < 0.05 vs. low-turnover bone disease.

### Association between urinary albumin excretion and bone histomorphometric parameters

In univariable analysis, higher urinary albumin excretion was associated with higher bone formation rate (BFR/BS). Given that bone turnover in CKD is influenced by kidney function and CKD-MBD biomarkers, this association was further examined in multivariable models adjusted for age, sex, eGFR, log₁₀-transformed iPTH, and log₁₀-transformed FGF23. In the primary complete-case multivariable model (*n* = 27), each 1-unit increase in log₁₀ urinary albumin excretion was associated with higher log₁₀-transformed BFR/BS [*B* = 0.295, 95% CI 0.088–0.501; *P* = 0.008], corresponding to an ∼1.97-fold higher BFR/BS. Coefficients for all covariates included in the primary multivariable model are provided in Supplementary Table S4. In sequential adjustment models using the same complete-case sample (*n* = 27), the association between urinary albumin excretion and BFR/BS remained statistically significant and the regression coefficient remained largely unchanged after incremental adjustment for eGFR, iPTH, FGF23, age, and sex (Supplementary Table S3). Variance inflation factors for variables included in the primary multivariable model ranged from 1.20 to 3.24, indicating no evidence of problematic multicollinearity. For the primary model, there was no evidence of departure from linearity (quadratic term, *P* = 0.562) or heteroscedasticity (Breusch–Pagan test, *P* = 0.541). No observation exceeded the conventional leverage threshold of 2p/n or a Cook’s distance of 1. Sensitivity analyses excluding potentially influential observations did not materially alter the association between urinary albumin excretion and BFR/BS. Higher urinary albumin excretion was also associated with osteoblast surface (Ob.S/BS) [*B* = 0.588, 95% CI 0.187–0.988; *P* = 0.005] and osteoclast surface (Oc.S/BS) [*B* = 0.464, 95% CI 0.041–0.887; *P* = 0.033]. Urinary albumin excretion was not significantly associated with osteoid surface, eroded surface, mineralization-related parameters, or bone volume (Table 3).

**Table 3:** Multivariable linear regression analyses of the association between urinary albumin excretion and bone histomorphometric parameters.

	B (95% CI)	*P* value
Turnover		
BFR/BS (µm^3^/µm^2^/yr)	0.295 (0.088–0.501)	0.008
Ob.S/BS (%)	0.588 (0.187–0.988)	0.005
Oc.S/BS (%)	0.464 (0.041–0.887)	0.033
OS/BS (%)	0.184 (−0.001–0.368)	0.051
ES/BS (%)	0.334 (−0.034–0.701)	0.074
Mineralization		
Mlt (d)	0.014 (−0.118–0.146)	0.825
OV/BV (%)	0.200 (−0.002–0.403)	0.052
O.Th (µm)	0.031 (−0.034–0.096)	0.338
Volume		
BV/TV (%)	0.037 (−0.022–0.096)	0.207

Urinary albumin excretion and histomorphometric variables were log₁₀-transformed before analysis. Models were adjusted for age, sex, estimated glomerular filtration rate, log₁₀(iPTH), and log₁₀(FGF23). Regression coefficients represent the expected change in the log₁₀-transformed histomorphometric parameter per 1-unit increase in log₁₀ urinary albumin excretion. Models were fitted using complete cases; *n* = 27 for BFR/BS and Mlt, and *n* = 41 for all other histomorphometric parameters. See Table 2 for abbreviations.

The pattern of adjusted associations across remodeling-related histomorphometric parameters is summarized in Fig. 1. Overall, the primary association with BFR/BS was accompanied by exploratory associations with cellular indices of bone turnover, whereas associations with other turnover-related parameters did not reach statistical significance. Sensitivity analyses using total urinary protein excretion as the exposure variable are presented in Supplementary Table S1. These analyses showed a partly overlapping pattern: total urinary protein excretion was associated with BFR/BS and Ob.S/BS, but not with Oc.S/BS; it was also associated with eroded surface and osteoid-related indices. Additional exploratory sensitivity analyses restricted to the primary histomorphometric outcome showed that the association between urinary albumin excretion and BFR/BS was consistent after further adjustment for diabetes mellitus, serum phosphorus, 25(OH)D, RAAS inhibitor use, systolic blood pressure, glomerular CKD etiology, or diabetic CKD etiology, each added separately to avoid model overfitting (Supplementary Table S2).

**Figure 1:**
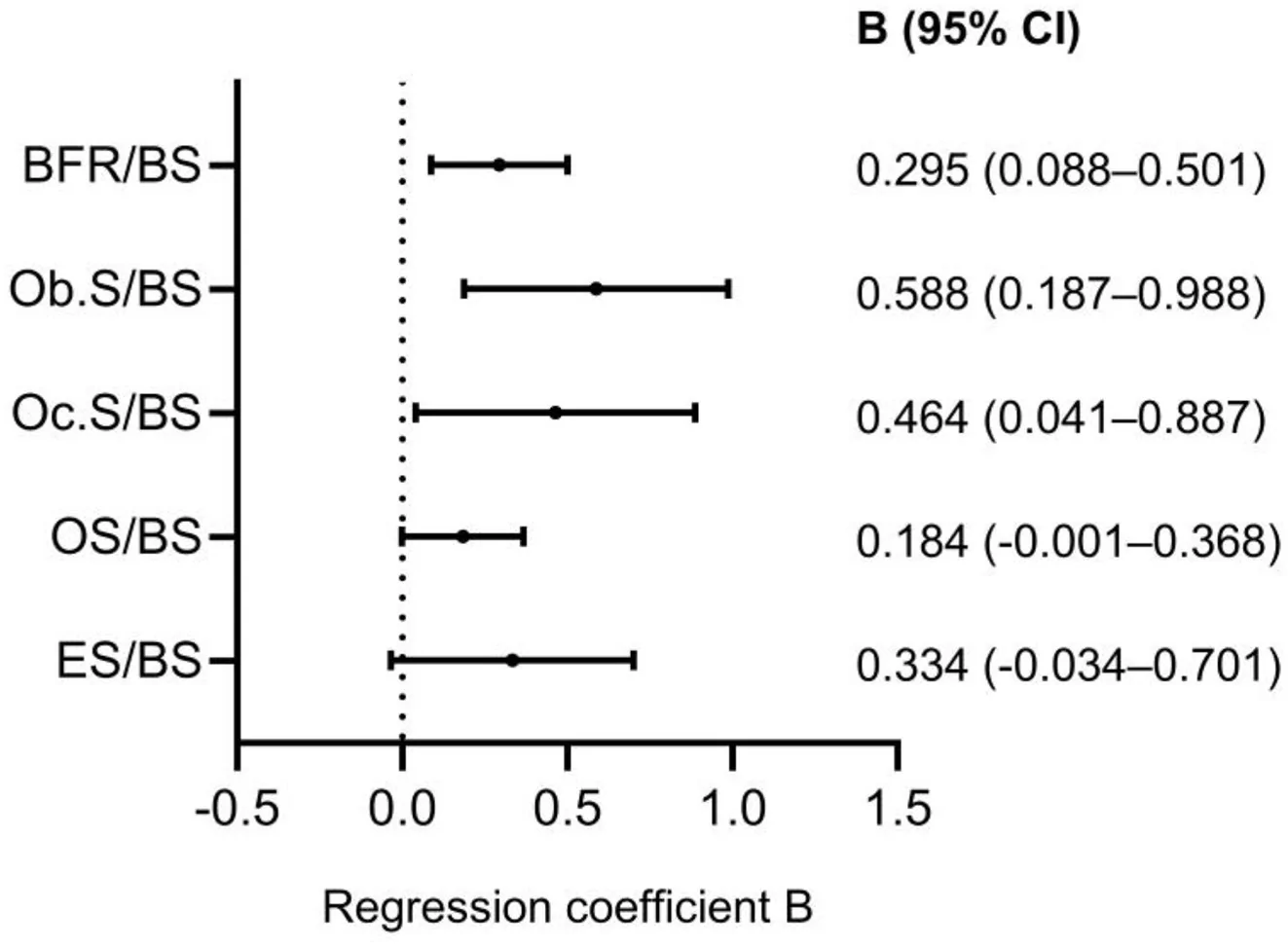
Forest plot of adjusted associations between urinary albumin excretion and turnover-related bone histomorphometric parameters. Points and horizontal lines represent regression coefficients and 95% CIs from multivariable linear regression models adjusted for age, sex, eGFR, log₁₀(iPTH), and log₁₀(FGF23). Urinary albumin excretion was log₁₀-transformed before analysis; coefficients represent the expected change in the log₁₀-transformed histomorphometric parameter per 1-unit increase in log₁₀ urinary albumin excretion. The vertical line represents the null value. BFR/BS was the prespecified primary turnover-related histomorphometric parameter; the remaining parameters were exploratory. Abbreviations are defined in Table 2.

## DISCUSSION

In this cohort of patients with CKD stages 3–4 who underwent transiliac bone biopsy, higher urinary albumin excretion was independently associated with BFR/BS, the prespecified primary turnover-related histomorphometric parameter. Exploratory analyses showed concordant associations with osteoblast surface and osteoclast surface, supporting a histomorphometric pattern consistent with increased bone turnover. This pattern was observed after adjustment for age, sex, eGFR, iPTH, and FGF23, suggesting that the association between albuminuria and higher bone turnover was not fully explained by filtration function or circulating CKD-MBD biomarkers included in our models. No statistically significant associations were observed with mineralization-related parameters or bone volume. To our knowledge, this is the first study to examine urinary albumin excretion in relation to quantitative bone histomorphometric parameters in predialysis CKD.

Albuminuria is a marker of glomerular injury and an independent predictor of CKD progression and cardiovascular outcomes [8–10]. Proteinuric kidney disease has also been linked to disturbances in mineral metabolism, including altered phosphate handling, vitamin D homeostasis, FGF23–Klotho signaling, and other manifestations of CKD-MBD [11–17]. Epidemiological studies have further associated albuminuria with adverse skeletal outcomes, including increased fracture risk [8, 9, 37–39]. Our findings extend these observations by providing direct quantitative histomorphometric evidence linking albuminuria to turnover-related bone remodeling indices.

Bone biopsy studies in nephrotic syndrome and primary glomerular diseases have described abnormalities of bone remodeling in the presence of heavy proteinuria [19, 20]. Experimental studies have shown that proteinuric serum can stimulate osteoblastic RANKL expression and promote osteoclastogenesis [21], whereas active vitamin D ameliorates bone abnormalities in experimental nephrotic syndrome [22]. These observations provide biological plausibility for the histomorphometric pattern observed in the present study. In this context, albuminuria should be interpreted as a potential marker of altered histomorphometric bone turnover rather than as a proven causal mediator. Although the association remained largely unchanged after sequential adjustment for eGFR, iPTH, and FGF23, residual confounding related to the severity of the underlying kidney disease or other unmeasured biological processes cannot be excluded. Whether this pattern contributes to the adverse skeletal outcomes linked to albuminuria requires confirmation in larger prospective cohorts [9, 37, 38].

Urinary albumin excretion was numerically highest in patients with high-turnover bone disease, but differences across histological categories did not reach statistical significance. This finding may reflect the loss of information inherent to categorizing continuous histomorphometric measurements, particularly in a biologically continuous disorder such as renal osteodystrophy [23, 40]. Accordingly, this descriptive finding should not be interpreted as evidence of a categorical association between albuminuria and high-turnover bone disease.

Sensitivity analyses using total urinary protein excretion provided partial support for the primary albuminuria findings but also highlighted differences between the two exposures. Total proteinuria reproduced the associations with bone formation rate and osteoblast surface, but not with osteoclast surface, and showed additional associations with eroded surface and osteoid-related indices. Given the limited sample size, however, these differences may reflect limited statistical power and sampling variability rather than genuine biological differences between albuminuria and total proteinuria and should therefore be interpreted cautiously. Similarly, exploratory sensitivity analyses for the primary outcome showed that the association between urinary albumin excretion and BFR/BS remained directionally consistent after additional adjustment for several clinical and biochemical covariates, each added separately to limit model overfitting.

Several methodological aspects and limitations should be acknowledged. The study used quantitative bone histomorphometry to assess individual histomorphometric parameters directly, rather than relying on circulating surrogate biomarkers. Adjustment for key determinants of CKD-MBD and the absence of prior exposure to medications known to directly affect bone metabolism reduced potential confounding. However, the cross-sectional design, modest sample size, and selected single-center biopsy cohort limit causal inference, precision, and generalizability. In particular, residual confounding by the severity of the underlying nephropathy or other unmeasured determinants of kidney injury cannot be excluded despite multivariable adjustment. In addition, the limited number of complete observations relative to the number of covariates in the primary multivariable model may reduce the precision and stability of the regression estimates and increase the risk of overfitting. The primary adjustment model was deliberately parsimonious to limit overfitting, and complete-case analyses precluded more extensive multivariable adjustment or formal subgroup analyses, including sex-stratified analyses. Given the number of correlated histomorphometric parameters evaluated, associations with cellular and secondary histomorphometric indices should be interpreted as exploratory.

In conclusion, in patients with CKD stages 3–4, higher urinary albumin excretion was associated with quantitative histomorphometric indices of higher bone turnover after multivariable adjustment, but not with mineralization-related parameters or bone volume. These findings suggest that albuminuria may mark a bone remodeling phenotype not fully captured by eGFR, iPTH, and FGF23. Larger prospective studies are needed to define the biological basis and clinical implications of this association.

## Supplementary Material

sfag346_Supplemental_Files

## Data Availability

The data underlying this article may be shared upon reasonable request to the corresponding author.
