# Supplementary material for: Albuminuria is associated with higher turnover-related bone histomorphometric indices in chronic kidney disease stages 3–4
Source: Clin Kidney J. 2026 Oct 1;19(10):sfag346. doi: 10.1093/ckj/sfag346 (PMC13655740; doi:10.1093/ckj/sfag346)
Supplement: Supplementary file 1 [file sfag346_supplemental_files.zip › Supplementary Table S1.docx]

Supplementary Table S1. Sensitivity analyses using total urinary protein excretion as the exposure variable in multivariable regression models

|  | | B (95% CI) | *P* value |
| --- | --- | --- | --- |
| Turnover | |  |  |
|  | BFR/BS (µm^3^/µm^2^/yr) | 0.433 (0.094 to 0.771) | 0.016 |
|  | Ob.S/BS (%) | 0.782 (0.175 to 1.389) | 0.013 |
|  | Oc.S/BS (%) | 0.375 (−0.285 to 1.035) | 0.257 |
|  | OS/BS (%) | 0.326 (0.069 to 0.582) | 0.015 |
|  | ES/BS (%) | 0.522 (0.008 to 1.035) | 0.048 |
| Mineralization | |  |  |
|  | Mlt (d) | 0.022 (−0.173 to 0.218) | 0.816 |
|  | OV/BV (%) | 0.314 (0.031 to 0.598) | 0.032 |
|  | O.Th (µm) | 0.223 (−1.053 to 1.499) | 0.726 |
| Volume | |  |  |
|  | BV/TV (%) | 1.108 (−2.570 to 4.786) | 0.540 |

Total urinary protein excretion was log₁₀-transformed before analysis. Variables with skewed distributions were analyzed after log₁₀-transformation; for histomorphometric variables containing zero values, 0.001 was added before transformation. Models were adjusted for age, sex, estimated glomerular filtration rate, log₁₀(iPTH) and log₁₀(FGF23). Regression coefficients represent the expected change in the dependent variable per 1-unit increase in log₁₀ total urinary protein excretion. These analyses were performed as sensitivity analyses; BFR/BS was the primary turnover-related histomorphometric parameter, and the remaining parameters were exploratory. Models were fitted using complete cases; n = 30 for BFR/BS and Mlt, and n = 45 for all other histomorphometric parameters. See Table 2 for abbreviations.
