# Supplementary material for: Albuminuria is associated with higher turnover-related bone histomorphometric indices in chronic kidney disease stages 3–4
Source: Clin Kidney J. 2026 Oct 1;19(10):sfag346. doi: 10.1093/ckj/sfag346 (PMC13655740; doi:10.1093/ckj/sfag346)
Supplement: Supplementary file 1 [file sfag346_supplemental_files.zip › Supplementary Table S2.docx]

Supplementary Table S2. Exploratory sensitivity analyses for the association between urinary albumin excretion and BFR/BS after additional covariate adjustment

| Model | B for log_10_ urinary albumin excretion | 95% CI | *P* value |
| --- | --- | --- | --- |
| Primary model | 0.295 | 0.088 to 0.501 | 0.008 |
| Primary model + diabetes mellitus | 0.330 | 0.123 to 0.537 | 0.003 |
| Primary model + serum phosphorus | 0.313 | 0.087 to 0.540 | 0.009 |
| Primary model + 25(OH)D | 0.299 | 0.071 to 0.528 | 0.013 |
| Primary model + RAAS inhibitor use | 0.295 | 0.082 to 0.509 | 0.009 |
| Primary model + systolic blood pressure | 0.265 | 0.068 to 0.463 | 0.011 |
| Primary model + glomerular CKD etiology | 0.280 | 0.061 to 0.498 | 0.015 |
| Primary model + diabetic CKD etiology | 0.298 | 0.080 to 0.516 | 0.010 |

BFR/BS, bone formation rate/bone surface; 25(OH)D, 25 hydroxyvitamin D; RAAS, renin-angiotensin-aldosterone system; CKD, chronic kidney disease.

Models were based on the primary multivariable model adjusted for age, sex, eGFR, log₁₀(iPTH) and log₁₀(FGF23). Each additional covariate was added separately to avoid overfitting. Urinary albumin excretion and BFR/BS were log₁₀-transformed before analysis. Regression coefficients represent the expected change in log₁₀(BFR/BS) per 1-unit increase in log₁₀ urinary albumin excretion. Glomerular CKD etiology and diabetic CKD etiology were entered as binary indicator variables. Models were fitted using complete cases; n = 27 for all models except the model additionally adjusted for 25(OH)D, which included 26 participants.
