# Supplementary material for: Albuminuria is associated with higher turnover-related bone histomorphometric indices in chronic kidney disease stages 3–4
Source: Clin Kidney J. 2026 Oct 1;19(10):sfag346. doi: 10.1093/ckj/sfag346 (PMC13655740; doi:10.1093/ckj/sfag346)
Supplement: Supplementary file 1 [file sfag346_supplemental_files.zip › Supplementary Table S3.docx]

Supplementary Table S3. Sequential adjustment models for the association between UAE and BFR/BS

| Model | Covariates included | Regression coefficient (B) for log_10_ UAE | 95% CI | *P* value |
| --- | --- | --- | --- | --- |
| Model 1 | Unadjusted | 0.248 | 0.071 to 0.425 | 0.008 |
| Model 2 | + eGFR | 0.238 | 0.054 to 0.421 | 0.014 |
| Model 3 | + eGFR + log₁₀(iPTH) | 0.285 | 0.115 to 0.455 | 0.002 |
| Model 4 | + eGFR + log₁₀(iPTH) + log₁₀(FGF23) | 0.287 | 0.105 to 0.468 | 0.003 |
| Model 5 | + eGFR + log₁₀(iPTH) + log₁₀(FGF23) + Age + Sex (primary model) | 0.295 | 0.088 to 0.501 | 0.008 |

BFR/BS, bone formation rate/bone surface; UAE, urinary albumin excretion; eGFR, estimated glomerular filtration rate; iPTH, intact parathyroid hormone; FGF23, fibroblast growth factor 23.
UAE and BFR/BS were log₁₀-transformed before analysis. Regression coefficients (B) represent the expected change in log₁₀(BFR/BS) associated with a 1-unit increase in log₁₀ UAE. Models were fitted sequentially by incrementally adding eGFR, log₁₀(iPTH), log₁₀(FGF23), age, and sex to the univariable model. All models were fitted in the same complete-case sample (n = 27) to ensure comparability across sequential models. Model 5 corresponds to the primary multivariable model reported in Table 3.
