# Supplementary material for: Albuminuria is associated with higher turnover-related bone histomorphometric indices in chronic kidney disease stages 3–4
Source: Clin Kidney J. 2026 Oct 1;19(10):sfag346. doi: 10.1093/ckj/sfag346 (PMC13655740; doi:10.1093/ckj/sfag346)
Supplement: Supplementary file 1 [file sfag346_supplemental_files.zip › Supplementary Table S4.docx]

Supplementary Table S4. Full coefficients of the primary multivariable linear regression model for BFR/BS

| Variable | B | 95% CI | *P* value |
| --- | --- | --- | --- |
| log₁₀ urinary albumin excretion | 0.295 | 0.088 to 0.501 | 0.008 |
| Age | 0.003 | −0.016 to 0.022 | 0.742 |
| Sex | 0.037 | −0.377 to 0.450 | 0.856 |
| eGFR | 0.030 | −0.015 to 0.074 | 0.180 |
| log₁₀(iPTH) | 0.858 | 0.118 to 1.598 | 0.025 |
| log₁₀(FGF23) | 0.007 | −0.671 to 0.685 | 0.984 |

BFR/BS, bone formation rate/bone surface; eGFR, estimated glomerular filtration rate; iPTH, intact parathyroid hormone; FGF23, fibroblast growth factor 23. Urinary albumin excretion, BFR/BS, iPTH and FGF23 were log₁₀-transformed before analysis. B values are unstandardized regression coefficients. The model included 27 participants with complete data for all variables.
